# Chitosan-Based Nanomaterials for Drug Delivery

**DOI:** 10.3390/molecules23102661

**Published:** 2018-10-16

**Authors:** Jianghua Li, Chao Cai, Jiarui Li, Jun Li, Jia Li, Tiantian Sun, Lihao Wang, Haotian Wu, Guangli Yu

**Affiliations:** 1 Key Laboratory of Marine Drugs, Ministry of Education & Shandong Provincial Key Laboratory of Glycoscience and Glycotechnology, School of Medicine and Pharmacy, Ocean University of China, Qingdao 266003, China; jianghuali0225@163.com (J.L.); 13573204095@163.com (J.L.); lijun@stu.ouc.edu.cn (J.L.); lijia199208@163.com (J.L.); 13070807917@163.com (T.S.); wlh418135925@163.com (L.W.); biochemiwu@gmail.com (H.W.); glyu@ouc.edu.cn (G.Y.); 2 Laboratory for Marine Drugs and Bioproducts, Qingdao National Laboratory for Marine Science and Technology, Qingdao 266237, China

**Keywords:** chitosan, nanomaterials, drug delivery

## Abstract

This review discusses different forms of nanomaterials generated from chitosan and its derivatives for controlled drug delivery. Nanomaterials are drug carriers with multiple features, including target delivery triggered by environmental, pH, thermal responses, enhanced biocompatibility, and the ability to cross the blood-brain barrier. Chitosan (CS), a natural polysaccharide largely obtained from marine crustaceans, is a promising drug delivery vector for therapeutics and diagnostics, owing to its biocompatibility, biodegradability, low toxicity, and structural variability. This review describes various approaches to obtain novel CS derivatives, including their distinct advantages, as well as different forms of nanomaterials recently developed from CS. The advanced applications of CS-based nanomaterials are presented here in terms of their specific functions. Recent studies have proven that nanotechnology combined with CS and its derivatives could potentially circumvent obstacles in the transport of drugs thereby improving the drug efficacy. CS-based nanomaterials have been shown to be highly effective in targeted drug therapy.

## 1. Introduction

Drug administration is generally performed to treat disease progression, except for autoimmune regulation [1]. An ideal drug should be capable of reaching a specific lesion and accumulating to its effective concentration [2]. Abundant approaches have been developed and utilized for drug administration, and the most commonly used method has been proven to be oral administration, which has been shown to be highly efficient, induces minimal side effects, and is easy to administer [3,4]. In addition, injection administration of substances, such as small molecules, proteins, or nucleic acids, have also been extensively employed via intravenous, subcutaneous, intramuscular, and intra-arterial injections in the clinic [5]. Transdermal administration refers to the administration through a mucous membrane, including nasal and oral mucosal tissues [6]. This review describes different types of drug administration to improve drug efficacy. However, drug transport has been associated with major obstacles due to the intricacy of the circulatory system, the complicated digestive system, and the powerful immune system in various organisms [7,8]. When a drug enters the body, it potentially fails to achieve the desired effects, owing to its short half-life, rapid clearance, and inability to reach the disease site [9,10]. In addition, the substance could be hydrolyzed in the stomach when it interacts with gastric acids [11]. Biological drugs, such as proteins and nucleic acids, are also probably digested by specific enzymes in vivo [12]. Various studies have described methods in circumventing these problems of drug delivery, including the use of nanomaterials as drug transport vehicles [13,14]. 

Nanotechnology has produced an extremely important impact on nano-biomedicine and the diagnosis/treatment of disease [15]. Nanomaterials in drug delivery can improve the cellular uptake of poorly soluble drugs [16,17]. They also enhance drug bioavailability at effective doses that were previously difficult to achieve. Studies have shown that both nanoparticle geometry and size play important roles in drug delivery [18,19]. Nanoparticle geometry could significantly affect the rate of drug loading and drug transport, and the balance between the size and geometry is critical to the efficiency of drug delivery. In addition, drugs wrapped by nanomaterials prolong their circulation time, thereby improving their biodistribution and pharmacodynamics [4,20]. Lastly, nanomaterials modified by other bioactive molecules would exhibit distinct functions, including specific targeting or the characteristics of environmental responsiveness, thereby facilitating in drug accumulation and better reaching its effective dose [21,22]. 

**Figure 1:**
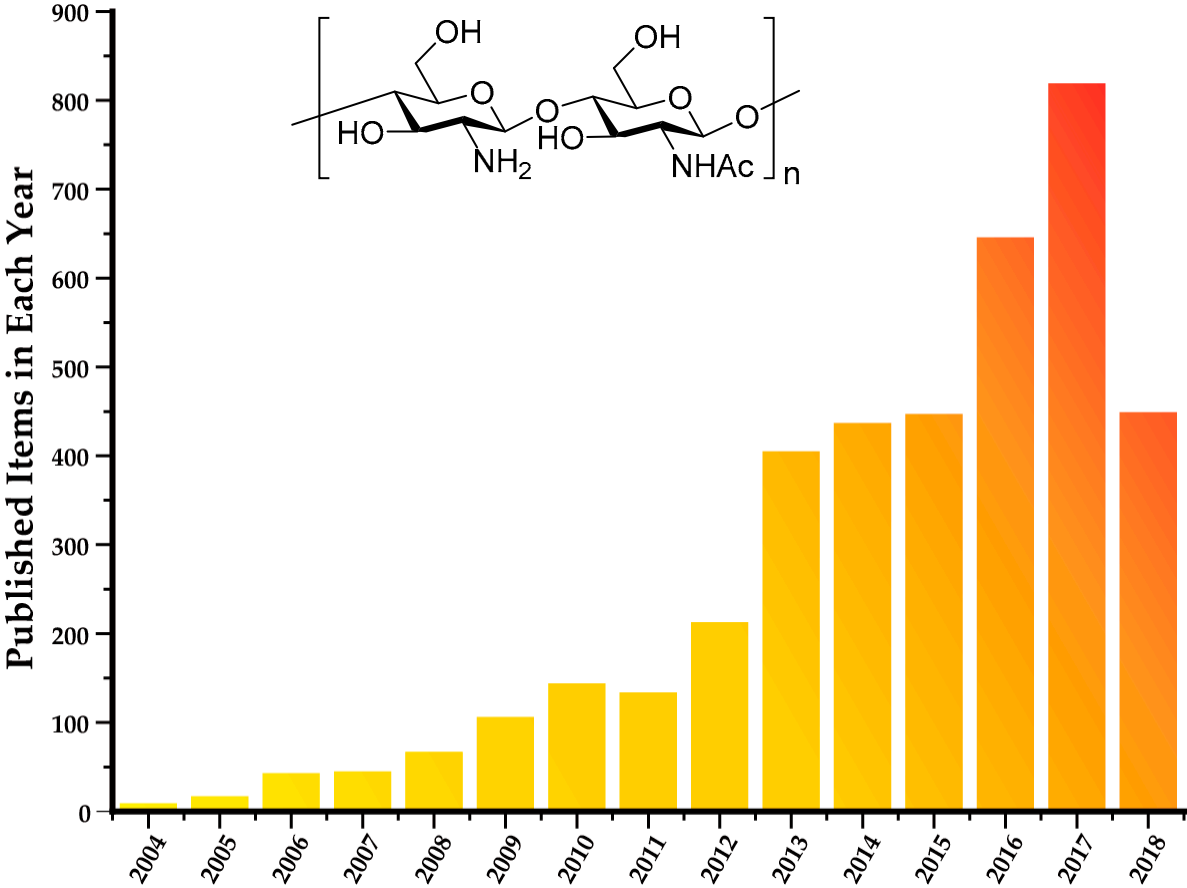
Number of articles published by years on chitosan-based nanomaterials for drug delivery. Data from Web of Science until 10 September 2018.

Chitosan (CS), a biological and cationic polysaccharide, has been considered as a promising nanomaterial with extensive medical applications [23,24]. CS is one of the most abundant biopolymers derived from natural chitin that commonly exists in the exoskeletons of arthropods, crustacean shells, insects, and fungal cell walls [25]. Structurally, CS is a representative polysaccharide with native amine groups that are positively charged. It consists of randomly distributed β-(1 → 4)-linked *N*-acetyl-d-glucosamine (GlcNAc) and d-glucosamine (GlcN) (Figure 1), and the presence of GlcN imparts its cationic character at neutral/physiological pH [26,27,28]. It possesses unique properties such as nontoxicity, biocompatibility, biodegradability, bioactivity, and mucoadhesion [29,30]. CS can be degraded by internal enzymes, such as lysozymes and chitosanases, to acquire oligosaccharides and monosaccharides, which are subsequently absorbed by the body [31]. Despite its unique physicochemical and biological properties, CS has not been extensively utilized in the clinic due to its low solubility and poor mechanical properties [32,33]. However, various approaches for modifying CS have been developed to address these issues [34,35]. Free amino and hydroxyl groups have been utilized to generate a wide range of CS derivatives with improved solubility based on its high affinity with functional proteins and the capability to self-assemble [36,37]. Thus, CS has been widely employed in various biomedical and pharmaceutical processes, such as drug/gene/vaccine delivery, tissue engineering, wound healing, and manufacture of cosmetic products [38,39,40]. Over the past decade, novel nanomaterials successfully developed from CS have been increasingly reported (Figure 1), with potential applications to targeted drug delivery based on the following features: (1) biocompatibility and ability to serve as reaction sites with other bioactive compounds, (2) protecting unstable drug molecules from strong gastric acids and blood flow responses, (3) ability to adhere to mucosal tissues to improve the absorption of specific drugs, (4) ease in combining with anionic biomacromolecules such as DNA by electrostatic action, and (5) colon-targeted administration [11,41,42]. Some reviews about chitosan-based nanoparticle systems for disease treatment have been published and they talk about biological applications of chitosan [43,44]. The present review comprehensively discusses recent advances in the development of the versatile nanomaterials derived from CS and its derivatives for well-controlled drug delivery.

## 2. Approaches in Generating CS Derivatives

**Scheme 1:**
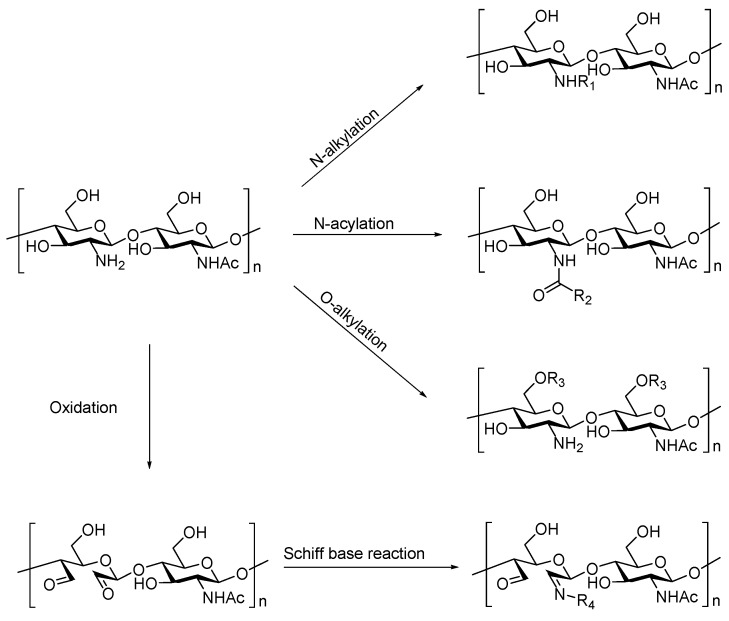
The basic reaction types of chitosan modification.

Chitosan derivatives mainly originate from the modification of hydroxyl and especially the free amino of chitosan skeleton [45]. The chitosan decorated with different functional groups yields desired products with excellent properties, and the effort about chitosan modification has given much assistance for its wide use in biomedical field [17]. For the binding to chitosan amino, the carboxyl group or acyl chloride on specific molecules form amide bonds with the amino group of chitosan, and sometimes alkylation occurs on the amino group of chitosan skeleton. For the modification of the hydroxyl, there are also many related cases that will be discussed later. Schiff base reaction sometimes is used to attach the carbonyl of the oxidized chitosan to the specific molecules with amino groups [46]. In briefly, the basic reaction types of chitosan structure modification are *N*-acylation, *N*-alkylation, *O*-alkylation, and oxidation-Schiff base reaction (Scheme 1)

### 2.1. General Modifications of CS to Improve Solubility

CS only dissolves under acidic conditions and shows poor solubility under natural humoral or intracellular environments at a pH of 6.8 to 7.4, thereby significantly limiting its applications to drug delivery [34,47]. Because of its poor solubility, CS cannot be modified by other bioactive compounds that are generally difficult to directly absorb and undergo normal blood circulation [48]. Therefore, the primary purpose for modification of natural CS is to improve its solubility (Table 1).

### 2.2. CS-Based Modifications for Targeted Drug Delivery

When a drug enters the human body, various obstacles prevent it from reaching the disease site to elicit a specific therapeutic effect [54]. More importantly, drug often impart severe toxic side effects [55]. The current most effective strategy for solving the issues above mentioned is targeted drug delivery [2,56]. CS derivatives harboring functional molecules can enhance their ability to target specific organs due to their high affinities to the organ’s surface [57]. The human body is a huge factory built with complicated materials and cross-linked signals between different types of organs and cells, and the considerable efforts have been devoted on studying the profound mystery in association with human signaling [58,59]. Previous studies have established various receptor and donor relationships in the body that can be developed for drug targeting delivery [60,61]. A wide variety of substances can be used for targeted organ delivery, including peptides, sugars, and other biological molecules [62]. Wen et al. reported that the combination of trimethyl chitosan (TMC) and ligand CSKSSDYQC (CSK) together (CSK-TMC) can be successfully used to treat breast cancer by oral administration. It has also been proven that the CSK-TMC conjugate performs a better antitumor therapeutic effect compared to free drug solution due to the selective affinity of CSK to intestinal cells [63]. According to Amiji et al., epidermal growth factor receptor (EGFR) was overexpressed in the human lung carcinoma A549 cell line, and EGFR-targeted nanoparticles have been developed from CS with a PEG linker that silenced the essential mitotic checkpoint gene Mad2 alone or in combination with cisplatin at subtherapeutic doses [64]. Yin et al. developed estrone-modified glycol chitosan nanoparticles (GCNP-ES) for breast cancer treatment because estrogen receptor (ER) is overexpressed in breast cancer tissues compared to normal mammary glands [65]. The conjugated estrone of the nanoparticles could significantly increase drug accumulation in tumor sites, thereby enhancing therapeutic effect. In addition, under a low pH tumor microenvironment, it is now possible to graft glycol chitosan with pH-responsive poly(2-(diisopropylamino) ethyl methacrylate) (PDPA), a hydrophobic substrate at physiological pH 7.4, which becomes hydrophilic at pH values below 6.3 due to the protonation during pH-triggered drug release. Other sophisticated and well-designed examples based on CS for targeted drug delivery are listed in Table 2.

### 2.3. CS-Based Modifications to Improve Environmental Responsiveness

The internal environment of the human body is complex and not static, and the environment of the lesion will also affect the development of the diseases [83]. The pH [7], cytokine [60], enzymes [5], ion concentration, and cell membrane pore size may vary in different tissues and organs to some extent. The pH of the pure gastric juice is 1–1.5, the pH of the physiological environment is about 7.4, and the tumor extracellular microenvironment is 6.5, pH of endosomes and lysosomes is about 5.0–6.2 and 4.0–5.0, respectively [84,85]. Therefore, the pH distribution in the human body supplies an excellent triggering condition for drug delivery and release. Simultaneously, different types of enzymes and cytokine would increase or decrease in acute and chronic arthritis, and the temperature at the site of inflammation is slightly higher [86]. Scientists have designed a series of precise environmental (e.g., pH, enzyme, and temperature)-responsive drug delivery vehicles based on the human body [87,88]. The attachment of pH-responsive molecules to CS achieves better responsive characteristics and facilitates drug delivery and release according to the environmental response [17]. In addition, amino acids are important pH-sensitive compounds. The charge state of amino acids depends on the environmental pH because these are amphoteric compounds containing both acidic and basic groups, and changes in pH can reverse the charge of an amino acid from positive to negative [89]. Zhang et al. developed a pH-sensitive nanogel by combining *N*-lysinal-*N′*-succinyl chitosan (NLSC), poly(*N*-isopropylacrylamide) (pNIPAA), and bovine serum albumin [90]. Based on the pH characteristics of the tumor microenvironment, NLSC that was designed with pH sensitivity has an isoelectric point of pH at 6, which was achieved by attaching a lysine and succinic anhydride to the CS backbone. The pH sensitive nanogel would swell in the acidic environment while the pH value is lower than that of NLSC. The anticancer drug encapsulated in the nanogel would then be triggered to be rapidly released, and the smart nanogel would shrink back under neutral condition. 

Other environmentally responsive macromolecules such as polyacrylic acid embedded in chitosan can also serve as drug carriers with pH-responsive property [67]. A typical example is the controlled release of 5-fluorouracil (5-FU) that has its surface imprinted with CS nanomaterial. Wang et al. prepared a novel oral pH-sensitive colon-specific 5-FU delivery system by attaching poly(methyl methacrylate) (pMMA) to chitosan (CS-pMMA) based on the persulfate initiation of the surface graft polymerization [91]. CS-pMMA and 5-FU were employed as matrix microspheres and template molecule respectively to prepare5-FU imprinted pH-sensitive polymer. The drug delivery system thus involved the combined features of pH responsiveness and time-dependent release mechanism. Severe gastrointestinal reactions and side effects can be avoided by transferring the 5-FU to release at the site of colon and increase the concentration of the drug at the lesion, thereby overcoming the outflow of 5-FU into the stomach and the small intestine. In addition to the distribution characteristic of pH, changes in enzyme expression levels can also be exploited in the development of environment-responsive drug delivery systems. The tetrapeptide (Gly-Phe-Leu-Gly, GFLG) is effectively cleaved by cathepsin B (CAB), which is overexpressed in various cancer cells. Yu et al. introduced the GFLG to CS-based carrier and achieved the responsive release of loaded drugs, which realized the multi-functional transport integrated with prodrug function, tumor targeting, and enzyme-trigger release [82]. The representative modifications of CS with environmental responsiveness are presented in Table 3.

### 2.4. Other Modifications of CS

There are numerous meticulous modifications used to build special CS drug delivery vehicles. Prodrug and fluorescent tracer are also applied to the design of CS-based delivery vehicle to generate improved drug delivery systems [61]. Prodrugs are compounds obtained by chemical modification of biomolecules or drug delivery vehicles and may be inactive or less active in vitro and exhibit pharmacological effects by enzymes or acids in vivo to release the active drug [98]. The main purpose of using a prodrug is to improve the bioavailability, increase the stability, reduce the side effects of the drug and prolong the drug cycle time in the blood [42]. 

Multidrug resistance (MDR) is a major obstacle in anticancer drug delivery, and the P-glycoprotein (P-gp) as a multidrug resistance-associated protein (MRP) is commonly overexpressed in cancer cell membranes. The MRP pumps out the anticancer drugs with broad spectrum and maintains low drug levels in the cytoplasm. Indomethacin (IDM) has been demonstrated to inhibit MRP pumping and glutathione-S-transferase activities, and thus it is possible to enhance the cytotoxicity of doxorubicin (DOX) and vincristine. Kima et al. prepared a chitosan oligosaccharide (CSO)-indomethacin (IDM) conjugate (CI) via amide bond to fabricate chemosensitizing nanoparticles (NPs) for tumor-targeted drug delivery [99]. The chemosensitizing nanoparticles (NPs) formed in the self-assembly mode and IDM were grafted to the CSO backbone as hydrophobic groups. The chemosensitizing NPs effectively ameliorated the anti-proliferation potentials of DOX in MRP-expressed A549 cells. According to the study from Jiang et al., a novel chitosan-graft-metformin (CS-MET) prodrug was developed with high intracellular sensitivity to stimuli [46]. The drug MET and oxidative CS were combined by imine reaction for type 2 diabetes (T2D) therapy. The CS-MET could serve as a gene delivery vector and anti-diabetes prodrug, which would collapse to release the MET and gene due to proton pumping effects in the body. Similarly, Giannotti et al. designed a highly versatile polyelectrolyte complex to improve the enzyme replacement therapy of lysosomal storage disorders, and fluorescein molecules were attached to the modified CS backbone to improve drug delivery [66]. These studies show that modification of CS and its derivatives have greatly contributed to the wide exploration and application for targeted drug delivery in the developments of medical devices.

## 3. Different Methods of Preparing Nanomaterials Using CS and Its Derivatives

The development of nanomaterials is rapid, bringing unprecedented opportunities to the food, cosmetic, and pharmaceutical industries [10]. Different types of nanodrugs have distinct advantages in the field of medicine, serving as carriers in drug delivery with multi-functions such as targeting and sustainable release of specific drug [44,57]. This review mainly focuses on the CS-based nanomaterials that have been utilized in drug delivery. Based on the physiochemical characters of CS, proven approaches such as ionic gelation, covalently crosslinked, and self-assembly have been developed to fabricate different forms of nanomaterials. Furthermore, the combination of CS with special inorganic nanoparticles (mesoporous silica and magnetic and gold nanoparticles) has also been used for well-controlled drug delivery [20]. In addition, the nanomaterials of different sizes and morphology have been generated using methods earlier described. Hence, various protocols for the preparation of CS-based nanomaterials are discussed below in detail, and the alternative forms of nanomaterials and their applications are also summarized in Table 4.

### 3.1. CS-Based Self-Assembly

CS derivatives that possess hydrophilic functions is an excellent drug carrier with good biocompatibility, mucoadhesive property, and intestinal absorption into the body [100]. It is not feasible to deliver drugs by CS alone, thus biocompatible hydrophobic molecules are commonly designed to attach hydrophobic core for loading drugs [25]. Hydrophobic molecules such as natural lipids, cholesterol, and hydrophobic drugs are usually immobilized on CS based on *N*-acylation, *N*-alkylation, *O*-alkylation, and Schiff-base reactions as described above. The CS-based conjugates subsequently promote the formation of nanomaterials by self-assembly. 

Cheng et al. employed the TMC as raw material, which was subsequently conjugated with the oleyl-NHS via amidation [80]. The amphipathic oleyl-conjugated trimethyl chitosan (OTMC) was attached to other amphipathic compounds to form a favorable targeted gene delivery vehicle through self-assembly. Similarly, You et al. synthesized CS-stearic acid copolymer (CSO-SA) via reaction of the carboxyl groups of SA with the amino groups of chitosan oligosaccharide (CSO) in the presence of EDC, and the polyethylene glycol (PEG) with polypeptide was simultaneously linked to CSO [26]. The amphipathic CS derivatives were self-assembled into nanoparticles covered on the surface of gold nanospheres, which performed well in specific photothermal therapy to the tumors. In addition, Gan et al. modified CS using deoxycholic acid into nanoparticles (DNPs) for loading the insulin. Deoxycholic acid-modified nanoparticles could traverse the intestinal epithelium via the biomimetic bile acid pathway. Moreover, poly(lactic acid) (PLA) is a safe hydrophobic biodegradable polyester approved by the Food and Drug Administration (FDA). Chirachanchai et al. prepared chitosan whisker (CSWK) that natural CS was grafted with oligo (lactic acid) (OLA) to help therapeutic agents crossing the skin via a transdermal drug delivery mode [6]. The OLA performed as a hydrophobic core for loading hydrophobic drug lidocaine in this complex. 

### 3.2. Covalently Crosslinked Nanomaterials

Covalent cross-linking refers to the coupling of two or more molecules together via covalent chemical bonds [101]. Crosslinkers containing a plurality of functional groups such as organic dibasic acid or polyhydric alcohol play a critical role in the process of covalent cross-linking. Generally, the bridge bonds between the different polymer chains allow nanomaterials to form a three-dimensional structure using crosslinkers such as glutaraldehyde [102] and genipin [103]. It was also found that the three-dimensional structure of nanomaterials obtained by covalent cross-linking was much more stable.

Carbonnier et al. developed hollow CS/poly(acrylic acid) nanocapsules in size of sub 100nm for antibiotic therapy [104]. The gold nanoparticles was produced as sacrificial templates through sulfonated thiol groups and covered with chitosan and poly(acrylic acid) layer-by-layer via electrolyte interaction. Similarly, the beta lactam antibiotic amoxicillin was loaded through diffusion and electrostatic interactions. The gold core was subsequently hydrolyzed due to the presence of cyanide. Finally, glutaraldehyde was employed in the preparation of cross-linked capsules via Schiff-base formation. As an adjustable nanocapsules in size, their dispersity, concentration, and hollow size could be tuned by the gold nanoparticles and thick wall formed with the number of polyelectrolyte bilayers.

Soboyejo et al. designed prodigiosin encapsulated CS microspheres for targeting drug delivery [105]. Prodigiosin is a secondary metabolite of *Serratia marcescens* with the anti-cancer properties. The prodigiosin encapsulated chitosan microspheres was prepared via covalently cross-linking, and the glutaraldehyde played an important role as a cross-linker. It presented a positive proportion between the encapsulation efficiency and the drug/polymer ratio. Considering the experience results, the integrated prodigiosin contained CS nanospheres had a good effect to kill the breast cancer cell line.

### 3.3. Ionically Crosslinked Nanomaterials

CS is a natural carbohydrate with polycationic property and able to gel with negatively charged compounds or specific polyanionic molecules to form nanoparticles [106]. This gelation process is owing to the crosslinkages established by inter- and intramolecular interaction between cationic chitosan and these polyanions [41]. The positive characteristic of chitosan has been applied in the development of drug delivery vehicles. Sodium tripolyphosphate (TPP), an anionic compound, is the earliest and most common molecule developed for ionic crosslinking with CS. It was first reported by Bodmeier et al. for drug delivery in 1989 [101]. Moerschbacher et al. investigated the main factors affecting the formation of NPs using systematically statistical design of experiments (DoE) and found that the size of CS particles may be controlled based on its concentration, solvent environment, and the molar ratio of CS to TPP during the polymerization [107]. Mao et al. developed a core-shell nanocomposite for insulin delivery in oral administration utilized by intestinal mucus penetration [108]. The core was prepared by ionic crosslinking between polycationic CS and TPP, and insulin as a drug model was coated with the specific layer formed by electrostatic interactions. They developed this novel mucus-penetrating NP because most viruses with hydrophilic and neutral surface are less hindered during mucus diffusion. In addition, anionic macromolecules such as hyaluronic acid and alginate are also able to combine with cationic CS via electrolyte interaction to form the drug delivery nanomaterials. Tirelli et al. developed a synergic dendritic cells (DC) targeting nanomaterial. CD44, the major receptor for hyaluronic acid (HA), is a surface receptor that affects the maturation and adhesion of DCs and T-cell stimulation [79]. HA, an almost ubiquitous anionic glycosaminoglycan (GAG), interacts with cationic CS to form drug delivery nanocomposites. Simultaneously, because mannose has affinity to the extracellular lectin, and conjugates of HA and mannose increase the absorption of nanocomposites to DCs. Hence, they produced mannosylated HA-CS nanocomposites via polyelectrolyte complexation for DC targeting delivery. Similarly, Mertins et al. obtained the pH-sensitive nanovehicle by dripping the anionic alginate to the stirred and fully deacetylated CS solution for treatment of endoparasites [109]. The anthelmintic drug was loaded into the nanocarrier and its biological activity was tested in fish. Chu et al. developed a core-shell NP for programmed sequential drug release system by the polyelectrolyte interaction between anionic poly(lactic-co-glycolic acid) (PLGA) and CS [110]. The drug was respectively encapsulated in the lipophilic PLGA core and the hydrophilic CS shell. In the process of drug delivery, a part of the drug was first released rapidly to achieve the drug effect in the stomach due to the acidic sensitivity of CS. The remaining drug in the hydrophobic polylactic acid nucleus was slowly released, and this release process was in line with the healing mechanism of the human body. In addition, 6-phosphogluconic trisodium salt [38], poly-c-glutamic acid [111] and poly(acrylic acid) (PAA) [67] can play the similar function to the molecule ionically crosslinked with chitosan as an anionic polymer through polyelectrolyte interaction.

### 3.4. Combining CS with Inorganic Nanomaterials

Many nontoxic inorganic nanoparticles are developed for drug delivery because of their excellent and well-controlled physical and chemical properties [112]. They are the porosity of mesoporous silica [113], the photosensitive fever and luminescence of gold nanoparticles [22], the magnetic responsiveness of iron oxide [32], and the luminescence of carbon quantum dots [52] that providing greater convenience for drug delivery and disease diagnosis. However, some disadvantages limited their applications in the field of biomedical, and they may tend to aggregate under physiological conditions for their poor stability, potential side effects for non-targeting, and difficulty in modification [114]. These inorganic nanoparticles applied in drug delivery systems (DDSs) is mainly composed by an inorganic nanoparticles core and a multifunctional surface coating. CS is a good candidate for decorating the inorganic nanoparticles because of its excellent properties aforementioned. Meanwhile, mesoporous silica nanoparticles (MSNs) are promising candidates for drug delivery because they have excellent characteristics of good biocompatibility, mesoporous structure, and chemical stability. McNally et al. developed a dual targeting mesoporous silica NP for the treatment of tumor [115]. The NP consisted of a mesoporous silica core and a urokinase plasminogen activator ligand (UPA) modified with a CS shell. The silica core was convenient to load drug for mesoporous feature and the UPA was a lipid with the affinity to urokinase plasminogen activator receptor overexpressed in pancreatic cancer, while CS played the pH-sensitive function of releasing the drug in an acidic tumor microenvironment. The integrated NP performed well for pancreatic cancer targeted drug delivery. Magnetic nanoparticles (MNPs) applied in drug delivery system can induce a drug to the targeting disease site under an external magnetic field for their high magnetization values. Ji et al. designed a drug delivery system that has dual functions of cancer thermo-chemotherapy and intracellular imaging based on smart multifunctional magnetic NP [88]. They loaded the MNP Fe_3_O_4_ as well as the drug 5-FU in the mesoporous SiO_2_ core and encapsulated the core in the shell of thermosensitive polymer poly(*N*-isopropylacrylamide) (PNIPAM) and cationic CS. The smart delivery vehicle was designed to effectively release drug at 45 °C under tumor microenvironment. Moreover, they mounted fluorescent molecules on nanocarriers for cell imaging. The multi-responsive drug carriers showed outstanding advantages in targeting sustainable drug release and disease diagnosis. Gold nanospheres, a kind of photo-absorbing agents, are able to generate heat from optical light energy for photothermal antitumor therapy. You et al. prepared a multifunctional TNYL-peptide modified by chitosan-stearic acid copolymer (CSO-SA) and NP encapsulated with the hollow gold nanospheres (HAuNS) and near-infrared (NIR) fluorescent tracer [26]. The TNYL acted as a targeting molecule with high affinity to EpHB4 receptors on tumor cells. The treatment obviously congregated to EphB4-positive tumors and presented a dramatically antitumor effect under NIR laser irradiation. Carbon quantum dots (CQD) as novel nanomaterials have been widely studied for photothermal therapy and NIR fluorescence imaging in the field of biomedical applications because they possess unique optical properties of good biocompatibility, small size, low cost, near infrared (NIR) absorption and emission nature. Zhou et al. reported biocompatible hybrid nanogels with CS-carbon dots (CDs) by combining pH-sensitive CS and the fluorescent CDs into an integrated nanostructure for synergistic photothermal therapy [93]. The CDs hybrid nanogels (CCHNs) were nontoxic verified by the tests on cells and in vivo. It had a stable fluorescence for simultaneous near-infrared (NIR) imaging and presented an intelligent drug release in response to both NIR light and change in pH. Interestingly, Wu et al. developed a mitochondrial targeting fluorescent carbon quantum dot based on CS, they prepared the CQDs via a facile hydrothermal mixing of CS, ethylenediamine and mercaptosuccinic acid [116]. The synthesized CQDs had good properties such as low cost, easy for preparation, high mitochondria-targeting specificity, long-term photostability, and convenient surface modification. In addition, there are many inorganic materials that have been developed recently, including metal organic framework (MOF) [117] and layered double hydroxides (LDH) [118] cooperated with CS for the construction of DDSs.

## 4. Advanced Applications of CS-Based Nanomaterials for Drug Delivery

**Figure 2:**
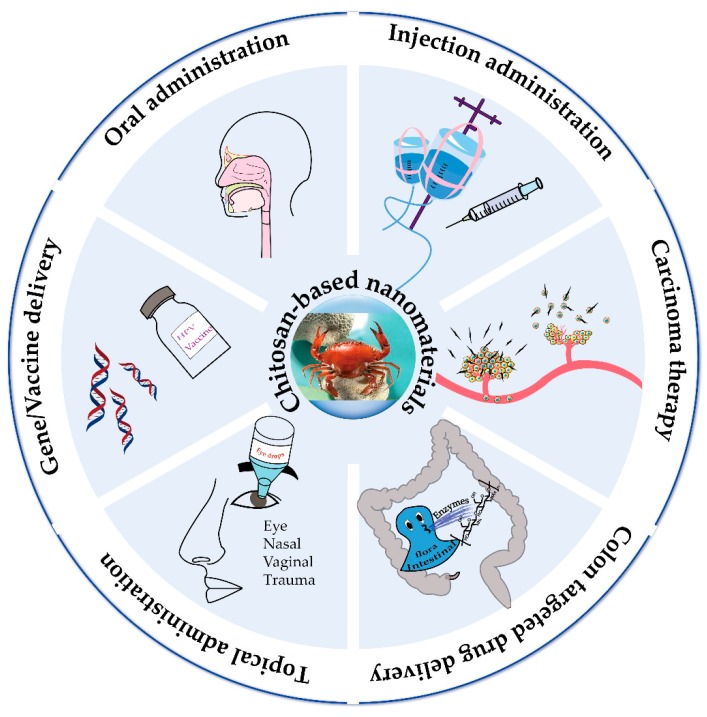
The advanced strategy for drug delivery based on CS nanomaterials.

The diseases disrupt normal physiological processes in the human body, thereby requiring effective treatment schemes [131]. The ideal drug should thus be safe, effective, stable, and specific. Drug delivery system should be designed based on their natural physical and chemical characteristics [22]. Different approaches have been used for the preparation of drug-loaded nanomaterials with natural or synthetic biocompatible compounds. Nanodrugs could be passively transported into cells, simultaneously they directly interact with lesion due to the presence of large area of surface. Nanomaterials also can be attached on different functional groups to target specific cells/tissues or stimulate responsiveness to release drug [132]. Meanwhile, specific strategies are designed and applied in the formation of nanomaterials for solving the disadvantages and obstacles in the process of drug transportation (Figure 2).

### 4.1. Oral Drug Delivery

Oral administration (OD) is the preferred dosage form for the development of new drugs because of its convenience, safety, and patient tolerance [3]. Drugs are absorbed into the blood circulation through the gastrointestinal epithelial cells after reaching the gastrointestinal tract, then their therapeutic effects are imparted to tissue and organs [20,133]. However, the OD method of drug administration is generally slow, and some drugs may be damaged by gastric acid or irritate the gastrointestinal tract [134]. Therefore, drugs for OD should be stable and do not induce gastric problems.

Insulin, the protein hormone secreted by Islet beta cells in the pancreas, is capable of decreasing blood glucose levels and promoting the synthesis of glycogen, fats, and proteins [23]. Patients usually administer insulin by subcutaneous (SC) injection daily or multiple times a day, which leads to healing difficulty of the skin damage due to multiple injections and their diabetic physique [135]. It is highly desirable to take insulin orally, although this route has a number of obstacles. Insulin permeability across biofilms is generally poor due to its hydrophilicity and high molecular weight [136]. In addition, insulin is readily inactivated in the stomach by gastric acids. Therefore, scientists are committed to develop oral delivery system of insulin for better treatment of diabetes [12]. Insulin is an anionic protein that is convenient to combine with CS via ionic interactions. Chen et al. developed a uniform core-shell NP via electrostatic complexation to deliver insulin by OD. *N*-(2-Hydroxy- propyl)-3-trimethylammonium chloride was modified with chitosan (HTCC) and insulin as positively charged core, whereas thiolated hyaluronic acid (HA-SH) acts as anionic shell [49]. The particles had an average size of 100 nm and performed well in encapsulation efficiency and loading capacity, thereby rendering it as a promising strategy for the delivery of insulin in diabetes, which have been well supported by in vitro and ex vivo experiments. Anderson et al. designed closed-loop insulin injectable delivery nanocapsules that were sensitive to glucose [137]. The nanocapsules were composed of pH-responsive chitosan matrix, glucose-specific enzymes and insulin via electrostatic complexation. The microgel system would swell to release insulin at higher rate under hyperglycemic conditions due to the enzymatic conversion of glucose into gluconic acid and CS-based protonation. The dual pH and enzyme response carrier was confirmed to smoothly regulate blood sugar levels in type 1 diabetic mice.

Gastric cancer, a commonly diagnosed cancer, exhibits malignant tumor originating from the gastric mucosa [138]. A polyphenol (−)-epigallocatechin-3-gallate (EGCG) extracted from green tea has been proven to inhibit tumor growth by antiangiogenesis, proliferation inhibition, and apoptosis induction. However, it is difficult to apply the EGCG to cure gastric cancer due to its low mucosal permeability and instability in the gastrointestinal environment. To overcome this problem, Feng et al. developed targeted NPs encapsulated with EGCG [81]. These nanomaterials are composed of three components including the fucose-conjugated chitosan (fucose-chitosan; FCS), PEG-conjugated chitosan (PEG-chitosan; PCS) complexes and gelatin (Gel), respectively. Fucose is a deoxyhexose sugar as a targeting molecular for its ability to modify various types of molecules physiologically. Chitosan enhances the gastrointestinal absorption via electrostatic interaction with abundant *N*-acetylneuraminic acid in gastric mucus, whereas polyethylene glycol (PEG) functions as a crosslinker, prolonging protein circulation in blood. It was found that the EGCG-loaded NPs were more effective against gastric tumors in vivo and in vitro.

In addition, Santos et al. designed a multifunctional tailorable composite system to deliver peptide drugs in oral based on the mucoadhesive chitosan [139]. Horcajada et al. developed a CS-coated mesoporous metal-organic frameworks (nanoMOFs) as a promising oral delivery vehicle. [117]

### 4.2. Injection Drug Delivery

Injection administration usually refers to injecting substances by intravenous, subcutaneous, intramuscular, and intra-arterial injections. Intravenous injection that directly injecting drugs into blood flow can ensure that all given drugs enter the systemic circulation and work quickly. Intramuscular or subcutaneous injections pass through layers of biofilm to reach the systemic circulation, with the feature of low bioavailability and slow efficacy. Arterial injection is mainly used for blood transfusion for severe illness. The chitosan nano-drugs are administrated by the parenteral injection to achieve therapeutic effects. 

Zhang et al. developed stepwise pH-responsive nanoparticles to release doxorubicin on demand [140]. The nanoparticles contained pH responsive dimethylmaleic acid and urocanic acid, and the DOX-loaded NPs could stepwise response to extracellular and intracellular pH after being injected to the tumor bearing mice. The DOX-loaded NPs was signifcantly uptake by tumor tissues in the slightly acidic tumor extracellular environment. Then the endo/lysosome acidic environment elicited the DOX release on-demand from NPs. The accumulation of DOX in tumor tissue was considerably better in amount and velocity for the stepwise pH-responsive NPs than the free DOX. Furthermore, they developed stepwise pH-/reduction-responsive nanoparticles for controlled DOX release via the tail vein injection [86]. Yan et al. designed CS-modified selenium nanoparticles (SC) to deliver TNF-α-derived peptide for improving the antitumor activity [39]. In the nanoparticles, SC was the slow-release carrier conjugated to the TNF-α-derived peptide P16 (SCP). The SCPs had a significant inhibitory effect especially on DU145 prostate cancer cells and were delivered through the tail vein injection. Zhang et al. developed the codelivery of DOX/TLR4 siRNA polymeric micelle with dual pH/redox sensitivity for cancer therapy [87]. Chitosan and polyethylenimine (PEI) were connected via disulfide bonds, and urocanic acid was decorated on the polymer covalently via amide bonds. Then the Toll-like receptor 4 siRNA (TLR4−siRNA) and DOX were packaged into the micelle, which were injected through the tail vein and performed well in the tumor therapy.

Injection administration possess obvious advantages in emergency situations because it works faster for severe or sudden illness. However, injection administration may cause some drawbacks including vascular injury, skin damage, serious bacterial and viral infection [141]. Thus, it is necessary to develop safer, more effective and sophisticated delivery systems [142].

### 4.3. Topical Drug Delivery

Topical medication can avoid systemic side effects caused by conventional oral and injection administration [143]. It can also reach the disease site through mucous membrane and skin penetration more quickly and directly [144]. The onset of the diseases occurred on the mucosa-rich parts included eyes, nasopharynx, mouth, rectum, urethra, vagina and bladder are more likely to use topical treatment [145]. CS, a natural cationic glycan with mucoadhesive property, has been widely applied to develop the advanced nanomaterials for topical drug delivery. 

Ophthalmic diseases are generally treated using topical instillation of active compounds. However, the method of treatment has been hindered by the inherent defense function of the eye. Drugs are thus degraded by metabolic enzymes in the ocular tissues. Yang et al. reported a curcumin-loaded lipid carrier for ophthalmic delivery [33]. They covalently modified *N*-acetylcysteine (NAC) on CS via amidation reaction between the primary amino groups of CS and the carboxylic acid group of NAC. The modification improved the solubility and performance of the CS vehicle. Cysteine is a compound containing a thiol group that may form disulfide bonds between molecules and tissues and the interaction prolongs the drug retention time. There are many sulfhydryl residues in proteins and mucus on the surface of tissues that may interact with thiol groups in the modified CS vehicle. CS played an important role as mucoadhesive component while the modified thiol group enhanced the effect of bioadhesion and precorneal retention. It has been proven to be a smart strategy that evidently prolong the residence time between the drug and the corneal. Shi et al. developed a novel block polymer composed of cationic CS and methoxy poly(ethylene glycol)-poly(ε-caprolactone) (MPEG-PCL) to deliver diclofenac in ocular diseases [123]. They attached the MPEG-PCL block polymer onto chitosan (MPEG-PCL-CS) by covalent coupling. The block polymer could self-assemble into cationic micelles and encapsulate hydrophobic drugs. The cationic micelles prolonged pre-corneal retention by electrostatic interactions with the negatively charged mucin and improved the bioavailability of the drug by mucoadhesion of cationic CS. The prepared nanomaterial has been shown to perform well in ophthalmic delivery. 

Nasal administration is an alternative method of topical administration for anti-inflammatory, antibacterial, nasal congestion or hemostasis therapy [146]. More importantly, nasal administration can even pass the blood-brain barrier (BBB) that has been proven to conveniently direct drug delivery from the nose to the brain [17]. Intranasal delivery is a non-invasive route with improved safety and patient compliance. Katsarov et al. formulated a chemically cross-linked CS microparticles for nasal administration [147]. They prepared versatile particles by covalently crosslinking CS with different concentrations of glutaraldehyde and the mucin adsorption ability was compared subsequently. All the particles were proved to have a high affinity for the mucin in vitro and significantly extended the drug release time. The mucoadhesive CS-based microsphere is a promising novel delivery system for nasal administration.

### 4.4. Colon-Targeted Drug Delivery

The intestine harbor various types of microorganisms that produce a variety of enzymes degrading compounds with specific structures such as polysaccharides containing β-glycosidic bonds [148]. CS, a high affinity substrate to be decomposed by intestinal flora, is randomly distributed with β-(1 → 4)-linked GlcNAc and GlcN. Hence, CS is an ideal candidate for targeted colon drug delivery. Opanasopit et al. developed a pH-sensitive CS polymer to encapsulate curcumin (CUR) for colon-targeted drug delivery [31]. They compared the delivery capabilities of two CS-based carrier *N*-naphthyl-*N*,*O*-succinyl chitosan (NSCS) and *N*-octyl-*N*,*O*-succinyl chitosan (OSCS). CUR was loaded by dialysis that is a physical entrapment approach. The morphology of the micelles obviously changed in the different pH medium, and the release characteristics of CUR were found to be pH-dependent. The release of CUR significantly increased at GI tract pH levels. Taghdisi et al. reported an epirubicin (Epi) encapsulated in CS-modified PLGA NPs via physical adsorption [128]. Interestingly, they modified 5TR1 DNA aptamer on the surface of nanomaterial to enhance the anti-tumor efficacy. The targeted and pH-sensitive nanoparticle presented better therapeutic effects. This strategy was proved to possess great potential in medical applications. Situ et al. prepared CS-based particles via ionic crosslinking [134]. They encapsulated proteins into the granules by crosslinking to maintain protein vitality during storage and oral administration. Particle morphology, protein loading, and release under different conditions were evaluated indicating the particles could effectively target the colon.

### 4.5. Carcinoma (Tumors) Therapy

Carcinoma involves the uncontrolled proliferation of cells. Surgery and chemotherapy usually are combined as a comprehensive strategy to conquer the tumor [149]. Chemotherapy is a systemic treatment with the advantage of eliminating remaining potential metastatic lesions after surgery. It has the advantage to treat multiple lesions simultaneously but the disadvantage is also apparent due to the systemic side effects that may affect healthy tissue [150]. Various optimized chemotherapy strategies have been developed to solve abovementioned issues. Nanomaterials have been designed to target specific tissues or respond to particular environmental conditions. CS is generally developed to antitumor nanovehicles for the treatment of carcinoma because of its unique properties such as mucoadhesiveness and structural variability.

Yin et al. developed hydrophilic NPs with cationic TMC co-delivered of DOX and interleukin-2 (IL-2) for enhanced antitumor efficacy [95]. *Cis*-aconitic anhydride was used to covalently graft DOX onto TMC to form nanocomplex with release of DOX in a pH-sensitive pattern. IL-2 was combined with NPs via electrostatic adsorption without compromising its bioactivity. Folate (FA) has also been loaded on nanocarrier for targeted delivery. The sub-spherical nanocomplexes have an average size about 200 nm and are positively charged. The optimized combination therapy strategy showed improved antitumor efficacy and reduced in vivo size effects. 

Shen et al. prepared integrated formulations combined DOX and Roussin’s black salt (RBS), a photosensitive nitric oxide (NO) into engineered nanospheres for tumor therapy [151]. NaYF4:Er upconversion nanoparticles (UCNPs) are currently the most efficient up-conversion luminescence matrix material that are excited by low-energy light and emitting high-energy light. UCNPs have been capped with the long-chain active carboxylic acid and then linked to oleoyl-CS using *N*-hydroxysuccinimide. The encapsulated DOX is released by pH-responsive swelling of the nanospheres. NO release is triggered by the encapsulated UCNPs and RBS, and the synergistic therapeutic effect demonstrated it was an effective method for cancer therapy.

Murugan et al. designed a combinatorial nanocarrier for amalgamation of anti-tumor agents in breast cancer cells and introduced the drug topotecan (TPT) and quercetin (QT) into a single system [67]. They prepared the mesoporous silica nanoparticles (MSNs) in a typical reaction and obtained a MSN-NH_2_ as white powder. The MSN core was then loaded with TPT and then covered with poly (acrylic acid) (PAA) and CS as shell, and conjugated with QT on the surface of TPT loaded in the MSN core. Finally, the arginine-glycine-aspartic acid (cRGD) was attached to the integrated drug-containing NPs, which targeted to cancer cells via integrin receptor-mediated endocytosis. Drug release was triggered by inter-tissue/intracellular pH change and CS degradation. This strategy has been shown to be effective in breast cancer treatment.

There are many publications about chitosan-based nanomaterials for antitumor drug delivery in innovative ways [152,153]. Effective drug delivery systems are developed for anti-cancer therapy based on environmental response and targeting principles to deliver drugs, vaccines, etc. Moreover, the drug delivery vectors were also designed with combination of photodynamic and hyperthermia therapy [112,125]. 

### 4.6. Gene Delivery

Genes encoded specific proteins are essential for various physiological processes of the body and their mutation often results in disease [11]. Gene therapy is a promising strategy to treat genetic diseases. Gene therapy refers to curing genetic diseases via the introduction of a foreign gene into a target cell to correct or repair a damaged gene. However, DNA and RNA molecules can be destroyed by harsh acids and enzymes that are produced in the body. In addition, DNA and RNA are anionic polymers which have good affinity with cationic polymers such as CS [154]. 

Small interfering RNA (siRNA) is a double-stranded RNA of 20 to 25 nucleotides in length and has many important functions in biology [155]. It primarily plays a unique role in the RNA interference events which regulates gene expression in a specific manner. siRNA technology has been widely used in various disease areas such as tumors and inflammation. However, it is rarely used in the clinic due to its poor stability affected by countless nucleases in vivo [156].

Stride et al. developed a CS-deoxycholic acid nanodroplets loading on the magnetic nanoparticle for siRNA delivery [127]. They covalently grafted deoxycholic acid on the CS and sequentially had perfluoropentane and iron oxide modified on the surface. siRNA combined on the surface of the particle via electrostatic interactions with a cationic CS matrix. The integrated siRNA and nanoparticles displayed stable in serum at 37 °C for up to 4 h. Interestingly, the nanodroplets would undergo a phase change under the assistance of ultrasound from a liquid state to a microbubble state with a larger volume and higher energy and release the contained siRNA, and the iron oxide-containing NP may be directed to the correct site while applying an external magnetic force. Importantly, treatment efficacy was positive correlated the energy of these emissions that were produced by ultrasound. Tumor protein p53, an unequal protein, has been shown to respond to cellular stresses, thereby acting as a tumor suppressor. Activating the proliferation suppressor gene may thus be a promising strategy for tumor therapy. Lin et al. achieved magnetic thymine-imprinted chitosan nanoparticles (TIPs) to active the expression of tumor suppressor p53 gene [129]. They prepared the MNPs using the Massart’s method. Then, the TIPs were developed via precipitating CS and separately mixing the thymine and MNPs. The thymine and telomeric DNA sequence exhibited high affinity to cationic CS. It was confirmed that the imprinted NPs present a high expression of p53 gene at the cellular level. Feron et al. developed noncovalent PEGylated CS nanoparticles for siRNA targeted delivery [68]. siRNA was loaded via electrostatic interaction with cationic CS. Targeting function was achieved by RGD peptides analog, and PEG would detach from the nanocarriers when necessary to facilitate cellular entry. Notch1 is a signaling receptor that plays a critical role in the rheumatoid arthritis (RA). Kim et al. developed a Notch1-targeting siRNA delivery nanoparticles (siRNA-NPs) to study anti-inflammatory effects [157].

### 4.7. Vaccine Delivery

Vaccine refers to biological products applied to prevent or control the occurrence and spread of infectious diseases [84]. The nature of the vaccine may be microbes or their toxins, enzymes, human or animal serum and cells. The emergence of vaccines has made a significant contribution to the prevention and control of diseases. However, there are many problems affecting the quality of the vaccine during the preparation, storage, and administration [158]. It is greatly necessary to take high quality control during the preparation of vaccines, because the inferior vaccines pose a great threat to human health. Nanomaterial-encapsulated vaccines are thus promising vaccine transport vehicles. CS is a particularly attractive choice for vaccine delivery because of its low immunogenicity, low toxicity, biocompatibility, and biodegradability [159]. Here we will discuss the gene delivery studies based on CS.

Tumor cell lysates can act as antigens to stimulate dendritic cells to produce immunity. Kong et al. developed tumor cell lysate-loaded (TCL) CS nanovaccine to enhance antitumor immunity by targeting dendritic cells (DCs) [78]. They modified chitosan with mannose (Man) for specific DCs targeting and then loaded TCL (Man-CTS-TCL NPs) to trigger an immune response. For the vaccine viability experiment, the efficacy of Man-CTS-TCL NPs as cancer vaccine was evaluated in vitro and in vivo. Specifically, the activation of DCs by Man-CTS-TCL NPs was studied at the cellular level, and the mice were challenged with B16 melanoma cells after they were vaccinated with the prepared vaccine. The experiment results showed the Man-CTS-TCL NPs are effective anti-tumor vaccines.

Tirelli et al. developed a dual targeting vaccine vector. CS showed an electrolyte interaction with HA, which potentially played an important role to load the siRNA [79]. Simultaneously, the mannosylated modification on HA further improved the targeting effect. The interaction of mannose with lectin promoted the internalization of NPs when hyaluronic acid was compatible with CD44, a surface receptor that has been reported to affect DC maturation, adhesion. The construction of targeting nanocarriers based on CS matrix is an effective approach for gene delivery with enhancement of immunomodulation effects.

DC-based vaccination is extremely limited because it demands multiple injections and ex vivo surgery. Han et al. developed a CS-based NP that achieving immunomodulation in vivo for cancer immunotherapy [158]. The CS NP was prepared via ionic crosslinking under the integration of TPP, and the ovalbumin (OVA) was encapsulated as a model antigen. Experimental data showed that the integrated vaccine could effectively promote the maturation of DCs and significantly improve anti-tumor effects. 

## 5. Conclusions and Future Prospects

First and foremost, CS is a linear and cationic polymer obtained from degradation and *N*-deacetylation of chitin that abundantly exists in the world, such as crustacean shells in ocean and vertebrates on land. CS has been developed and utilized as a high-value biomacromolecule, particularly in terms of biomedical applications. CS possesses good biocompatibility and low toxicity, immunity, and biodegradability, indicating it may serve as an effective drug delivery vehicle. The CS backbone consists of multiple free amino and hydroxyl groups that could be used as active sites, and versatile approaches have been reported for the construction of CS-based nanomaterials, such as nanogels, NPs, micelles, liposomes, nanofibers, and nanospheres. Taking advantage of the nanomaterials derived from CS, the drug delivery system has been extensively studied for oral and injection administration, topical delivery, colon-targeted drug delivery, carcinoma therapy, vaccine and gene delivery. It is worth mentioning that the clinical administration of CS-based drug vectors is still limited due to their potential risks. In summary, this review highlights recent advances in the development of CS-based nanomaterials for drug delivery. With novel CS derivatives inspired by glycochemistry and vast innovative materials brought by nanotechnology, CS-based nanomaterials would be fully explored for their promising biomedical applications, such as tissue engineering, wound dressing, drug delivery, and cancer diagnosis in the near future.

## Figures and Tables

**Table 1:** The common examples of modification of chitosan.

Reaction Type	Chitosan Derivatives			
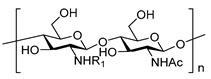 *N*-alkylation	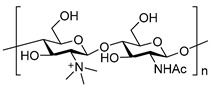	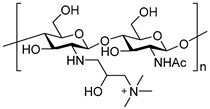		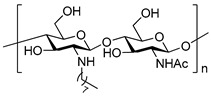
	Trimethyl chitosan [23] *N*-(2-hydroxy)-propyl-3-trimethyl chitosan [49] *N*-octyl chitosan [45]			
	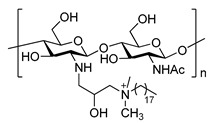		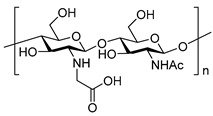	
	Octadecyl quaternization chitosan [50]		*N*-carboxymethyl chitosan [51]	
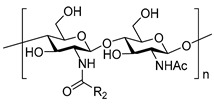 *N*-acylation	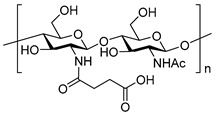		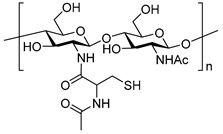	
	*N*-succinyl chitosan [31]		*N*-acetylcysteine chitosan [33]	
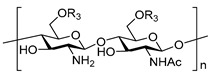 *O*-alkylation	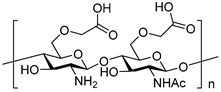		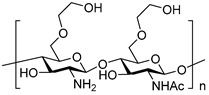	
	*O*-carboxymethyl chitosan [52]		Glycol chitosan [53]	

**Table 2:** Targeted drug delivery combined with chitosan.

Ligand Type	Ligand	Receptor/Targeting Site	Ref.
Peptide/protein	CSKSSDYQC peptide	Goblet cells	[63]
	EGFR-specific peptide	Asialoglycoprotein receptor	[64]
	Arginine-glycine-aspartic acid	Integrin α_v_β_3_	[66,67,68,69]
	TNYLFSPNGPIARAW peptide	EphB4	[26]
	Cell penetrating peptide	Intestinal mucosal layer	[70]
Vitamin	Folate (FA)	Folate receptor	[71,72]
	Vitamin B12	Epithelial cells	[73]
Hormone	Estrone	Breast cancer tissues	[65]
Carbohydrate	Lactobionic acid	ASGPR	[74]
	Lactose	ASGPR	[75,76]
	Galactose	ASGPR	[77]
	Mannose	Mannose receptor	[78,79,80]
	Fucose	Fucosylated abnormal cell	[81]
	Hyaluronic acid (HA)	CD44	[82]

**Table 3:** The representative modifications of chitosan with environmental responsiveness.

Triggering Conditions	Function	Grafting Molecule	Ref.
pH	Adjust the isoelectric point of the chitosan derivative to the expected point and reverse the charge under a certain pH condition. Simultaneously, keep the drug delivery system stable in the body fluid circulation and release the drug in response to the pH of the specific sites.	Arginine	[45]
		Lysine	[50]
		Vitamin B12	[73]
		Succinylation	[90,92]
		Gly-Phe-Leu-Gly (GFLG) tetrapeptide	[82]
		Ethylenediaminetetraacetic acid (EDTA)	[93]
		Dimethylmaleic anhydride (DMMA)	[94]
		*Cis*-aconitic anhydride (CA)	[95]
		Poly(2-(diisopropylamino)ethyl methacrylate)	[65]
		Poly(methyl methacrylate)	[91]
		Polyacrylic acid (PAA)	[96]
Temperature	Act as a hydrophobic core and release drug with temperature responsive.	pNIPAA	[88,90]
		Pluronics	[97]
Enzyme	Controlled drug release via structural collapse caused by enzymatic degradation.	GFLG peptide	[82]
Magnetic field	Direct delivery of the nanoparticle to specific site though external magnetic field.	Fe_3_O_4_	[88]

**Table 4:** The diverse nanomaterials with distinct nanomorphology formed by CS and its derivatives for drug delivery.

Morphology	The Role Chitosan Played	Preparation Method	Application	Ref
Nanogels	Chitosan-carbon dot hybrid nanogels	Covalent cross-linking	Photothermal−chemo therapy	[93]
	pH responsive eucalyptus oil coated double walled biodegradable nanogels	Ion crosslinking	Controlled drug delivery	[119]
	PEGylated and fluorinated chitosan nanogel	Covalent modification	Targeted drug delivery	[120]
	Reversible swelling-shrinking nanogel	Covalent modification/cross-linking	Character of deep tumor penetration	[90]
Micelles	Chitosan-based pH-sensitive polymeric micelles	Covalent modification/self-assembly	Colon-targeted drug delivery	[31]
	pH-responsive aerobic micelles	Ion crosslinking	Photodynamic therapy	[121]
	Chitosan-pluronic micelles	Covalent modification/self-assembly	Drug delivery for glioblastoma cancer	[122]
	Multifunctional nanoparticles	Covalent modification/self-assembly	Targeted photothermal therapy	[26]
	Chitosan grafted MPEG-PCL	Covalent modification/self-assembly	Ocular delivery of hydrophobic drug	[123]
Nanofibers	Biomimetic mineralization of carboxymethyl chitosan nanofibers	Electrospinning process	Improve osteogenic activity	[34]
Liposomes	Arginine-modified nanostructured lipid carriers	Covalent modification/self-assembly	Anticancer drug delivery	[45]
	Glycosaminoglycan modified chitosan liposome	Covalent modification	Antimalarial drug delivery	[124]
	Aptamer-modified liposomal complexes	Covalent modification/other processing	Reverse drug resistance in lung cancer	[125]
	Gold nanoshell-coated liposomes	Covalent modification/electrostatic adsorption	Photothermal and chemotherapy	[126]
	Glycol chitosan-coated liposomes	Covalent modification/self-assembly	pH-responsive drug-delivery	[35]
Nanosphere	Magnetic nanoparticle-loaded chitosan-deoxycholic acid nanodroplets	Covalent modification, self-assembly	siRNA Delivery	[127]
	Smart pH-responsive nanocarrier	Covalent modification/electrostatic adsorption	Targeted delivery of ursolic acid	[72]
	Thermoresponsive nanospheres	Covalent modification/emulsification/solvent evaporation method	Release drug for the treatment of osteoarthritis	[97]
Nano-particles	Uniform core-shell nanoparticles	Ion crosslinking	Enhance oral delivery of insulin	[49]
	*N*-trimethyl chitosan nanoparticles	Covalent modification/self-assembly	Oral delivery to treat breast cancer	[63]
	Chitosan-modified PLGA nanoparticles	Ion crosslinking	Tumor-targeted drug delivery	[128]
	EGFR-targeted chitosan nanoparticles	Covalent modification/self-assembly	SiRNA delivery	[64]
	Indomethacin-conjugated chitosan oligosaccharide nanoparticle	Covalent modification/self-assembly	Prodrug and tumor-targeted drug delivery	[99]
Inorganic nano-materials	Viable smart targeted nanoenvelope delivery system	Covalent modification/self-assembly	Dox encapsulated and targeted therapy	[71]
	Multifunctional magnetic nanoparticles	Covalent modification/ sonication treatment	Thermo-Chemotherapy Intracellular Imaging	[88]
	Combinatorial nanocarrier	Covalent modification/ion crosslinking	Drug delivery for breast cancer	[67]
	Magnetic thymine-imprinted chitosan nanoparticles	Physical adsorption	Gene therapy	[129]
	Functional hollow microspheres constructed from MOF shells	Covalent modification/Physical adsorption	Drug delivery and targeted transport	[130]
